# Crystal structure of 2,6-di­benzyl­pyrrolo­[3,4-*f*]iso­indole-1,3,5,7(2*H*,6*H*)-tetra­thione

**DOI:** 10.1107/S2056989017012154

**Published:** 2017-08-25

**Authors:** Hansu Im, Hyunjin Park, Tae Ho Kim, Chang Hwa Woo

**Affiliations:** a Department of Chemistry (BK21 plus) and Research Institute of Natural Sciences, Gyeongsang National University, Jinju 52828, Republic of Korea; b Director of Planning Center, Gyeongsang National University Academy and Industry Collaboration, 501 Jinjudaero, Jinjusi 52828, Republic of Korea

**Keywords:** crystal structure, pyromellitic di­imide, two-dimensional network, short S⋯S contact

## Abstract

The title compound, C_24_H_16_N_2_S_4_, consists of a central pyromellitic di­imide substituted with an S atom and terminal benzyl groups. The mol­ecule lies on a crystallographic inversion centre so that the asymmetric unit contains half of the mol­ecule. The mol­ecule was prepared by thio­nation of *N*,*N*′-di­benzyl­pyromellitic di­imide with Lawesson’s reagent and has an S-shaped conformation similar to other compounds of this type. The phenyl groups are tilted by 72.69 (8)° with respect to the plane of the central arene ring. In the crystal, mol­ecules are connected by C—H⋯π inter­actions and weak short S⋯S contacts, forming supra­molecular layers extending paralled to the *ab* plane. The crystal studied was found to be non-merohedrally twinned, with the minor component being 0.113 (3).

## Abstract 2

The title compound comprises a central pyromellitic di­imide substituted with S atoms and terminal benzyl groups. In the crystal, adjacent mol­ecules are linked by C—H⋯π inter­actions and short S⋯S contacts, forming a two-dimensional network parellel to the (110) plane.

## Chemical context   

Recently, pyromellitic di­imide derivatives have been spotlighted due to their use in energy-storage materials (Nalluri *et al.* 2016 ▸). They also show potential applications in photovoltaic devices (Kanosue *et al.*, 2016 ▸) and organic semiconductors (Zheng *et al.*, 2008 ▸). Not only pyromellitic di­imide derivatives, but also pyromellitic di­imides substituted with sulfur have potential applications in organic semiconductors (Yang *et al.*, 2015 ▸). We have reported copper(I) coordination polymers based on pyromellitic diimide derivatives (Park *et al.*, 2011 ▸), which showed colour change owing to intermolecular halogen–π interactions. In addition, we have found that reversible solvent exchange and crystal transformations were possible in the crystals (Kang *et al.*, 2015 ▸). In an extension of previous research, we have synthesized the pyromellitic di­imide in which the O atoms are replaced with S atoms, by the reaction of *N*,*N*′-di­benzyl­pyromellitic di­imide with Lawesson’s reagent, and report its crystal structure here.
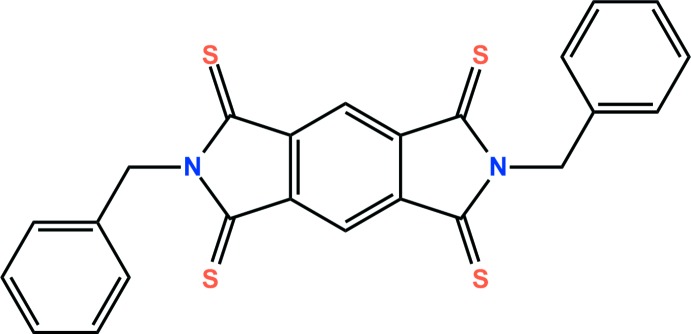


## Structural commentary   

**Figure 1:**
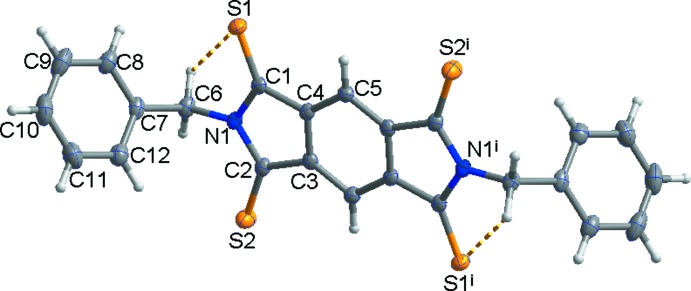
The asymmetric unit of the title compound, with displacement ellipsoids drawn at the 50% probability level. H atoms are shown as small spheres of arbitrary radius and yellow dashed lines represent intra­molecular C—H⋯S short contacts. Unlabelled atoms are generated by the symmetry operation (−*x* + 2, −*y* + 1, −*z*).

The mol­ecular structure of the title compound consists of a central pyromellitic di­imide substituted with S atoms and two terminal benzyl groups (Fig. 1 ▸). The mol­ecule possesses a crystallographic inversion centre and thus the asymmetric unit of the title compound is composed of half a mol­ecule. The mol­ecule exhibits an intra­molecular C6—H6*B*⋯S2 short contact (Table 1 ▸). In the mol­ecule, the terminal phenyl groups point in opposite directions and their planes are tilted by 72.69 (8)° with respect to the plane of the central arene ring, forming an elongated S-shaped mol­ecule.

## Supra­molecular features   

**Figure 2:**
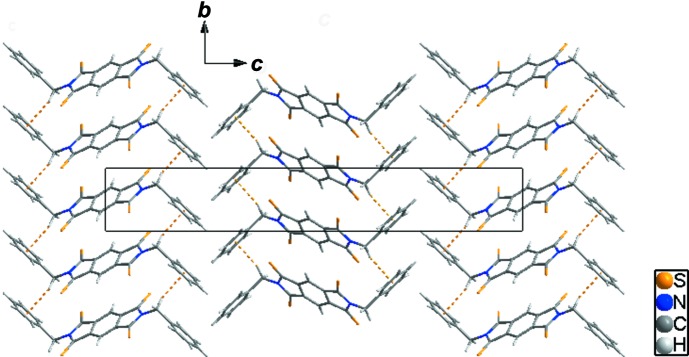
Inter­molecular C—H⋯π inter­actions (yellow dashed lines) forming one-dimensional loop chains.

**Figure 3:**
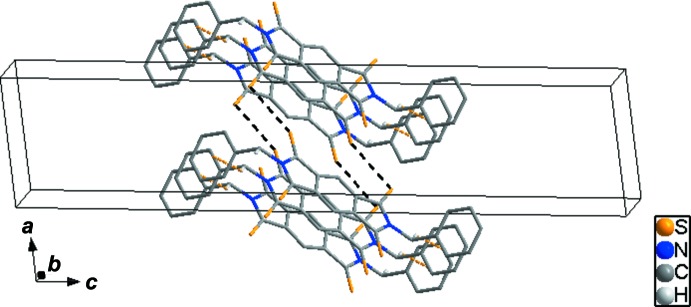
The packing diagram for the title compound, showing the two-dimensional network formed by C—H⋯π inter­actions (yellow dashed lines) and weak short S⋯S contacts (black dashed lines). H atoms not involved in inter­molecular inter­actions have been omitted for clarity.

In the crystal, C6—H6*B*⋯*Cg*1^i^ (*Cg*1 is the centroid of the C7–C12 ring) inter­actions between neighbouring mol­ecules generate a one-dimensional loop chain (yellow dashed lines in Fig. 2 ▸, and Table 1 ▸). Moreover, adjacent mol­ecules are connected by a weak short S1⋯ S2 contact [3.5921 (10) Å], resulting in the formation of a two-dimensional network (yellow and black dashed lines in Fig. 3 ▸).

## Synthesis and crystallization   

*N*,*N*′-Di­benzyl­pyromellitic di­imide was synthesized by the reaction of pyromellitic dianhydride with 2-phenyl­ethyl­amine according to the literature procedure of Im *et al.* (2017 ▸). To a stirred solution of *N*,*N*′-di­benzyl­pyromellitic di­imide (0.25 g, 0.63 mmol) in anhydrous toluene (100 ml) was added Lawesson’s reagent (2.00 g, 4.90 mmol), and the resulting mixture was stirred under reflux for 36 h. It was then cooled to room temperature and concentrated *in vacuo*, followed by purification by silica-gel flash column chromatography (CH_2_Cl_2_–*n*-hexane, 1:3 *v*/*v*). Crystals suitable for X-ray diffaction analysis were obtained by slow evaporation of a di­chloro­methane solution of the title compound.

## Refinement   

Crystal data, data collection and structure refinement details are summarized in Table 2 ▸. All H atoms were positioned geometrically and refined using a riding model, with C—H = 0.95 Å and *U*_iso_(H) = 1.2*U*_eq_(C) for aromatic C—H groups, and C—H = 0.99 Å and *U*_iso_(H) = 1.2*U*_eq_(C) for C*sp* ^3^—H groups. Non-merohedral twinning was identified in the crystal (TwinRotMat within *PLATON*; Spek, 2009 ▸); the twin law is −0.999 0 0.002, 0 −1 0, 1 0 0.999 and the final refined BASF parameter was determined to be 0.113 (3).

## Supplementary Material

Crystal structure: contains datablock(s) I, New_Global_Publ_Block. DOI: 10.1107/S2056989017012154/hg5493sup1.cif

Structure factors: contains datablock(s) I. DOI: 10.1107/S2056989017012154/hg5493Isup2.hkl

CCDC reference: 1570214

Additional supporting information:  crystallographic information; 3D view; checkCIF report

## Figures and Tables

**Table 1:** Hydrogen-bond geometry (Å, °) *Cg*1 is the centroid of the C7–C12 ring.

*D*—H⋯*A*	*D*—H	H⋯*A*	*D*⋯*A*	*D*—H⋯*A*
C6—H6*B*⋯S2	0.99	2.75	3.208 (3)	109
C6—H6*B*⋯*Cg*1^i^	0.99	2.66	3.498 (3)	142

Symmetry code: (i) 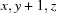.

**Table 2:** Experimental details

Crystal data	
Chemical formula	C_24_H_16_N_2_S_4_
*M*_r_	460.63
Crystal system, space group	Monoclinic, *P*2_1_/*c*
Temperature (K)	173
*a*, *b*, *c* (Å)	6.8571 (4), 4.7724 (3), 32.0010 (17)
β (°)	95.916 (4)
*V* (Å^3^)	1041.65 (11)
*Z*	2
Radiation type	Mo *K*α
μ (mm^−1^)	0.47
Crystal size (mm)	0.43 × 0.34 × 0.01

Data collection	
Diffractometer	Bruker APEXII CCD
Absorption correction	Multi-scan (*SADABS*; Bruker, 2014 ▸)
*T*_min_, *T*_max_	0.646, 0.746
No. of measured, independent and observed [*I* > 2σ(*I*)] reflections	1829, 1829, 1640
*R*_int_	0.059
(sin θ/λ)_max_ (Å^−1^)	0.595

Refinement	
*R*[*F* ^2^ > 2σ(*F* ^2^)], *wR*(*F* ^2^), *S*	0.038, 0.089, 1.05
No. of reflections	1829
No. of parameters	137
H-atom treatment	H-atom parameters constrained
Δρ_max_, Δρ_min_ (e Å^−3^)	0.26, −0.23

Computer programs: *APEX2* (Bruker, 2014 ▸), *SAINT* (Bruker, 2014 ▸), *SHELXS97* (Sheldrick, 2008 ▸), *SHELXL2014* (Sheldrick, 2015 ▸), *DIAMOND* (Brandenburg, 2010 ▸), *SHELXTL* (Sheldrick, 2008 ▸) and *publCIF* (Westrip, 2010 ▸).

## supplementary crystallographic information

### Crystal data

**Table:**

C_24_H_16_N_2_S_4_	*F*(000) = 476
*M**_r_* = 460.63	*D*_x_ = 1.469 Mg m^−^^3^
Monoclinic, *P*2_1_/*c*	Mo *K*α radiation, λ = 0.71073 Å
*a* = 6.8571 (4) Å	Cell parameters from 3915 reflections
*b* = 4.7724 (3) Å	θ = 2.6–25.5°
*c* = 32.0010 (17) Å	µ = 0.47 mm^−^^1^
β = 95.916 (4)°	*T* = 173 K
*V* = 1041.65 (11) Å^3^	Plate, brown
*Z* = 2	0.43 × 0.34 × 0.01 mm

### Data collection

**Table:**

Bruker APEXII CCD diffractometer	1640 reflections with *I* > 2σ(*I*)
φ and ω scans	*R*_int_ = 0.059
Absorption correction: multi-scan (SADABS; Bruker, 2014)	θ_max_ = 25.0°, θ_min_ = 1.3°
*T*_min_ = 0.646, *T*_max_ = 0.746	*h* = −8→8
1829 measured reflections	*k* = −5→5
1829 independent reflections	*l* = −6→38

### Refinement

**Table:**

Refinement on *F*^2^	0 restraints
Least-squares matrix: full	Hydrogen site location: inferred from neighbouring sites
*R*[*F*^2^ > 2σ(*F*^2^)] = 0.038	H-atom parameters constrained
*wR*(*F*^2^) = 0.089	*w* = 1/[σ^2^(*F*_o_^2^) + (0.037*P*)^2^ + 0.6197*P*] where *P* = (*F*_o_^2^ + 2*F*_c_^2^)/3
*S* = 1.05	(Δ/σ)_max_ < 0.001
1829 reflections	Δρ_max_ = 0.26 e Å^−^^3^
137 parameters	Δρ_min_ = −0.23 e Å^−^^3^

### Special details

**Table:**

Geometry. All esds (except the esd in the dihedral angle between two l.s. planes) are estimated using the full covariance matrix. The cell esds are taken into account individually in the estimation of esds in distances, angles and torsion angles; correlations between esds in cell parameters are only used when they are defined by crystal symmetry. An approximate (isotropic) treatment of cell esds is used for estimating esds involving l.s. planes.
Refinement. Refined as a 2-component twin

### Fractional atomic coordinates and isotropic or equivalent isotropic displacement parameters (Å^2^)

**Table:**

	*x*	*y*	*z*	*U*_iso_*/*U*_eq_	
S1	0.50612 (10)	0.30438 (16)	0.05735 (2)	0.0259 (2)	
S2	1.10897 (11)	0.99091 (17)	0.10558 (2)	0.0299 (2)	
N1	0.7913 (3)	0.6607 (5)	0.08677 (6)	0.0192 (5)	
C1	0.7132 (4)	0.4707 (6)	0.05665 (7)	0.0189 (6)	
C2	0.9678 (4)	0.7718 (6)	0.07722 (7)	0.0199 (6)	
C3	1.0046 (4)	0.6467 (6)	0.03656 (7)	0.0182 (6)	
C4	0.8521 (4)	0.4592 (6)	0.02468 (7)	0.0184 (6)	
C5	1.1572 (4)	0.6945 (6)	0.01231 (7)	0.0199 (6)	
H5	1.2604	0.8225	0.0204	0.024*	
C6	0.6888 (4)	0.7471 (6)	0.12291 (8)	0.0232 (6)	
H6A	0.5503	0.7888	0.1130	0.028*	
H6B	0.7493	0.9218	0.1348	0.028*	
C7	0.6944 (4)	0.5277 (6)	0.15724 (8)	0.0235 (6)	
C8	0.5208 (5)	0.4058 (6)	0.16745 (9)	0.0300 (7)	
H8	0.3993	0.4576	0.1525	0.036*	
C9	0.5252 (5)	0.2086 (7)	0.19950 (10)	0.0411 (9)	
H9	0.4069	0.1252	0.2064	0.049*	
C10	0.7023 (5)	0.1345 (7)	0.22125 (9)	0.0423 (9)	
H10	0.7052	0.0004	0.2433	0.051*	
C11	0.8741 (5)	0.2531 (7)	0.21127 (9)	0.0383 (8)	
H11	0.9952	0.2000	0.2263	0.046*	
C12	0.8711 (4)	0.4503 (6)	0.17935 (8)	0.0289 (7)	
H12	0.9901	0.5326	0.1726	0.035*	

### Atomic displacement parameters (Å^2^)

**Table:**

	*U*^11^	*U*^22^	*U*^33^	*U*^12^	*U*^13^	*U*^23^
S1	0.0227 (3)	0.0291 (4)	0.0261 (3)	−0.0078 (3)	0.0038 (3)	0.0008 (3)
S2	0.0311 (4)	0.0301 (5)	0.0291 (4)	−0.0100 (3)	0.0063 (3)	−0.0092 (3)
N1	0.0213 (11)	0.0168 (13)	0.0198 (10)	−0.0010 (10)	0.0041 (9)	0.0010 (9)
C1	0.0212 (13)	0.0160 (15)	0.0193 (12)	0.0008 (11)	0.0013 (10)	0.0051 (11)
C2	0.0235 (14)	0.0158 (16)	0.0210 (12)	0.0014 (12)	0.0046 (10)	0.0024 (11)
C3	0.0208 (13)	0.0153 (15)	0.0184 (12)	0.0035 (12)	0.0022 (10)	0.0026 (11)
C4	0.0196 (13)	0.0156 (16)	0.0202 (12)	−0.0001 (11)	0.0024 (10)	0.0046 (11)
C5	0.0208 (13)	0.0177 (16)	0.0209 (13)	−0.0005 (12)	0.0012 (10)	0.0021 (11)
C6	0.0279 (14)	0.0180 (17)	0.0249 (13)	0.0020 (12)	0.0089 (11)	−0.0020 (12)
C7	0.0359 (16)	0.0163 (16)	0.0197 (12)	−0.0004 (13)	0.0094 (11)	−0.0036 (11)
C8	0.0359 (16)	0.0259 (19)	0.0304 (15)	−0.0014 (14)	0.0145 (13)	−0.0022 (13)
C9	0.057 (2)	0.029 (2)	0.0427 (17)	−0.0066 (17)	0.0301 (16)	−0.0013 (15)
C10	0.070 (2)	0.032 (2)	0.0276 (16)	−0.0005 (18)	0.0169 (16)	0.0040 (14)
C11	0.053 (2)	0.033 (2)	0.0273 (15)	0.0034 (17)	0.0005 (14)	0.0030 (14)
C12	0.0365 (16)	0.0238 (18)	0.0272 (14)	−0.0037 (14)	0.0068 (12)	−0.0010 (13)

### Geometric parameters (Å, º)

**Table:**

S1—C1	1.629 (3)	C6—H6A	0.9900
S2—C2	1.635 (3)	C6—H6B	0.9900
N1—C2	1.384 (3)	C7—C12	1.388 (4)
N1—C1	1.390 (3)	C7—C8	1.394 (4)
N1—C6	1.473 (3)	C8—C9	1.390 (4)
C1—C4	1.469 (3)	C8—H8	0.9500
C2—C3	1.477 (3)	C9—C10	1.382 (5)
C3—C5	1.384 (4)	C9—H9	0.9500
C3—C4	1.399 (4)	C10—C11	1.374 (5)
C4—C5^i^	1.388 (3)	C10—H10	0.9500
C5—C4^i^	1.388 (3)	C11—C12	1.388 (4)
C5—H5	0.9500	C11—H11	0.9500
C6—C7	1.515 (4)	C12—H12	0.9500
			
C2—N1—C1	112.3 (2)	N1—C6—H6B	108.9
C2—N1—C6	124.4 (2)	C7—C6—H6B	108.9
C1—N1—C6	123.1 (2)	H6A—C6—H6B	107.7
N1—C1—C4	106.0 (2)	C12—C7—C8	119.4 (3)
N1—C1—S1	125.63 (19)	C12—C7—C6	120.6 (2)
C4—C1—S1	128.3 (2)	C8—C7—C6	120.0 (2)
N1—C2—C3	105.8 (2)	C9—C8—C7	120.1 (3)
N1—C2—S2	127.14 (19)	C9—C8—H8	120.0
C3—C2—S2	127.06 (19)	C7—C8—H8	120.0
C5—C3—C4	122.7 (2)	C10—C9—C8	119.7 (3)
C5—C3—C2	129.4 (2)	C10—C9—H9	120.1
C4—C3—C2	107.9 (2)	C8—C9—H9	120.1
C5^i^—C4—C3	122.5 (2)	C11—C10—C9	120.5 (3)
C5^i^—C4—C1	129.6 (2)	C11—C10—H10	119.8
C3—C4—C1	107.9 (2)	C9—C10—H10	119.8
C3—C5—C4^i^	114.8 (2)	C10—C11—C12	120.2 (3)
C3—C5—H5	122.6	C10—C11—H11	119.9
C4^i^—C5—H5	122.6	C12—C11—H11	119.9
N1—C6—C7	113.4 (2)	C11—C12—C7	120.1 (3)
N1—C6—H6A	108.9	C11—C12—H12	119.9
C7—C6—H6A	108.9	C7—C12—H12	119.9
			
C2—N1—C1—C4	−0.2 (3)	S1—C1—C4—C5^i^	−0.3 (4)
C6—N1—C1—C4	175.6 (2)	N1—C1—C4—C3	−1.2 (3)
C2—N1—C1—S1	−178.89 (19)	S1—C1—C4—C3	177.4 (2)
C6—N1—C1—S1	−3.1 (3)	C4—C3—C5—C4^i^	−0.3 (4)
C1—N1—C2—C3	1.5 (3)	C2—C3—C5—C4^i^	−180.0 (2)
C6—N1—C2—C3	−174.3 (2)	C2—N1—C6—C7	−108.7 (3)
C1—N1—C2—S2	−177.3 (2)	C1—N1—C6—C7	76.0 (3)
C6—N1—C2—S2	7.0 (4)	N1—C6—C7—C12	64.7 (3)
N1—C2—C3—C5	177.5 (3)	N1—C6—C7—C8	−116.7 (3)
S2—C2—C3—C5	−3.8 (4)	C12—C7—C8—C9	0.0 (4)
N1—C2—C3—C4	−2.2 (3)	C6—C7—C8—C9	−178.7 (3)
S2—C2—C3—C4	176.5 (2)	C7—C8—C9—C10	0.1 (5)
C5—C3—C4—C5^i^	0.3 (4)	C8—C9—C10—C11	−0.3 (5)
C2—C3—C4—C5^i^	−180.0 (2)	C9—C10—C11—C12	0.4 (5)
C5—C3—C4—C1	−177.6 (2)	C10—C11—C12—C7	−0.3 (5)
C2—C3—C4—C1	2.1 (3)	C8—C7—C12—C11	0.1 (4)
N1—C1—C4—C5^i^	−179.0 (3)	C6—C7—C12—C11	178.8 (3)

Symmetry code: (i) −*x*+2, −*y*+1, −*z*.

### Hydrogen-bond geometry (Å, º)

**Table:**

*D*—H···*A*	*D*—H	H···*A*	*D*···*A*	*D*—H···*A*
C6—H6*B*···S2	0.99	2.75	3.208 (3)	109
C6—H6*B*···*Cg*1^ii^	0.99	2.66	3.498 (3)	142

Symmetry code: (ii) *x*, *y*+1, *z*.
